# Pilicides inhibit the FGL chaperone/usher assisted biogenesis of the Dr fimbrial polyadhesin from uropathogenic *Escherichia coli*

**DOI:** 10.1186/1471-2180-13-131

**Published:** 2013-06-12

**Authors:** Rafał Piatek, Beata Zalewska-Piatek, Krystyna Dzierzbicka, Sławomir Makowiec, Justyna Pilipczuk, Kasjan Szemiako, Anna Cyranka-Czaja, Marek Wojciechowski

**Affiliations:** 1 Department of Microbiology, Gdańsk University of Technology, ul. Narutowicza 11/12, Gdańsk 80-233, Poland; 2 Department of Organic Chemistry, Gdańsk University of Technology ul. Narutowicza 11/12, Gdańsk 80-233, Poland; 3 Faculty of Biotechnology, University of Wroclaw, Tamka 2, Wrocław 50-137, Poland; 4 Department of Pharmaceutical Technology and Biochemistry, Gdańsk University of Technology ul. Narutowicza 11/12, Gdańsk 80-233, Poland

## Abstract

**Background:**

The global spread of bacterial resistance has given rise to a growing interest in new anti-bacterial agents with a new strategy of action. Pilicides are derivatives of ring-fused 2-pyridones which block the formation of the pili/fimbriae crucial to bacterial pathogenesis. They impair by means of a chaperone-usher pathway conserved in the Gram-negative bacteria of adhesive structures biogenesis. Pili/fimbriae of this type belong to two subfamilies, FGS and FGL, which differ in the details of their assembly mechanism. The data published to date have shown that pilicides inhibit biogenesis of type 1 and P pili of the FGS type which are encoded by uropathogenic *E. coli* strains.

**Results:**

We evaluated the anti-bacterial activity of literature pilicides as blockers of the assembly of a model example of FGL-type adhesive structures, – the Dr fimbriae encoded by a *dra* gene cluster of uropathogenic *Escherichia coli* strains. In comparison to the strain grown without pilicide, the Dr^+^ bacteria cultivated in the presence of the 3.5 mM concentration of pilicides resulted in a reduction of 75 to 87% in the adherence properties to CHO cells expressing Dr fimbrial DAF receptor protein. Using quantitative assays, we determined the amount of Dr fimbriae in the bacteria cultivated in the presence of 3.5 mM of pilicides to be reduced by 75 to 81%. The inhibition effect of pilicides is concentration dependent, which is a crucial property for their use as potential anti-bacterial agents. The data presented in this article indicate that pilicides in mM concentration effectively inhibit the adherence of Dr^+^ bacteria to the host cells, – the crucial, initial step in bacterial pathogenesis.

**Conclusions:**

Structural analysis of the DraB chaperone clearly showed it to be a model of the FGL subfamily of chaperones. This permits us to conclude that analyzed pilicides in mM concentration are effective inhibitors of the assembly of adhesins belonging to the Dr family, and more speculatively, of other FGL-type adhesive organelles. The presented data and those published so far permit to speculate that based on the conservation of chaperone-usher pathway in Gram-negative bacteria , the pilicides are potential anti-bacterial agents with activity against numerous pathogens, the virulence of which is dependent on the adhesive structures of the chaperone-usher type.

## Background

Bacterial pathogenesis is a complex process which has been well studied in the case of urinary tract infections (UTIs) mediated by uropathogenic *Escherichia coli* (UPEC) expressing type 1 and P pili. The crucial steps of this mechanism, namely, initial bacterial attachment, invasion and biofilm formation, are strictly dependent on the pili function [1,2]. These structures belong to the family of adhesive organelles assembled in accordance with the classical chaperone-usher pathway, which is highly conserved in Gram-negative bacteria. Pili, fimbriae or amorphic adhesive oganelles are linear homo- or heteropolymers of hundreds to thousands of protein subunits. All these proteins possess a conserved immunoglobuline-like structure denoted by the lack of the seventh β-strand, G. The effect of this structural defect is a hydrophobic acceptor cleft flanked by the β-strands A and F [3-6]. The folding of protein subunits is strictly dependent on the action of the specific periplasmic chaperone protein. The chaperone complements the defective structure of a subunit by donating a specific G1 donor β-strand in line with the donor strand complementation (DSC) reaction [5-8]. The stable chaperone-subunit complex migrates to the usher protein located in the outer membrane, where the process of protein subunit polymerization occurs. The formation of the functional adhesive organelle propagates in accordance with the donor strand exchange (DSE) reaction This step is dependent on the action of the N-terminal donor peptide exposed from each subunit [9-11].

Though global conservation of chaperone, usher and fimbrial proteins, the available structural data describing the assembly of different adhesive organelles, namely, P and type 1 pili of *E. coli*, F1 surface antigen of *Y. pestis*, Dr/Afa-III fimbriae of *E. coli*, SAF fimbriae of *S. typhimurium* and colonization factor CS6 of *E. coli*, also identify many important differences between them [12-14]. The specific structural and functional adaptations connected with the biogenesis of particular adhesive structures have been well studied in the case of chaperone and subunit proteins. Sequence and structural data comparisons allow the family of periplasmic chaperones to be divided into two subfamilies on the basis of the length of the loop connecting β-strand F1 with the donor G1 strand, the FGL and FGS subfamilies having a long and a short loop, respectively [15,16]. This loop is an important structural element which, in the chaperone-subunit complex, extends the acceptor cleft binding motif of the chaperone G1 donor strand. In the FGS chaperones, the β-strand G1 stabilizes a subunit core by donating only three bulky hydrophobic residues [4,7]. In the case of FGL chaperones, the G1 binding motif is typically extended by two additional, bulky, alternating hydrophobic residues from a loop region [5,13]. In the FGL chaperones, the second subunit-binding motif involved in the DSC mechanism is formed by three bulky hydrophopic residues located in the long N-terminal sequence forming the β-strand A1 [5,13]. The long F1-loop-G1 hairpin of these chaperones is stabilized by the disulfide bond conserved in the whole subfamily [17,18]. The longer G1 and A1 binding motif of the FGL chaperones correlates with the extended structure of the subunits’ acceptor cleft [13]. The molecular differences in the structure and function of the FGL and the FGS chaperones presented here correlate with the structure of the adhesive organelles which they assemble [13]. The FGL chaperones assemble organelles composed of only one type of protein subunit and, optionally, the second minor tip subunit [12,13]. They are characterized by a thin fimbrial, amorphous or capsule-like morphology. Each subunit of these homopolymeric structures possesses the host-cell receptor binding site or sites; thus, they are polyadhesins. In contrast, the FGS chaperones assemble heteropolymeric, well-structured adhesive pili composed of up to seven different subunits [10,19]. Pili are monoadhesins, as they possess only one receptor binding subunit located at the tip of the organelle. In addition, the division of chaperones and adhesive organelles into the FGS and FGL families also correlates with the phylogenetic analysis based on the usher ancestry. The FGL organelles belong to the γ3-monophyletic group, while the FGS can be divided into five clades: γ1, γ2, γ4, κ and π [20].

The adhesive organelles of the chaperone-usher type are unique virulence factors specific only to Gram-negative -pathogenic bacteria. The conservation of this mechanism renders it a good potential target for the development of antibacterial agents [21,22]. The pilicides originally proposed by Svensson *et al.* in 2001 are a class of low molecular weight agents, derivatives of a dihydrothiazolo ring-fused 2-pyridone scaffold which block formation of pili by affecting the function of chaperone [22]. According to the crystallographic and biological data, pilicides target the chaperone-usher pathway by blocking interaction between the N-terminal domain of the usher and chaperone-subunit complex. Hence, the pilicides block the formation of pili by preventing a DSE reaction. Pilicides bind to the hydrophobic patch of residues located in the F1, C1, D1 region of the N-terminal domain conserved in all chaperones [23]. This region encompasses part of the F1-G1 loop which is structurally rearranged during the formation of the chaperone-subunit complex (DSC reaction). The dynamic nature of this region is also reflected in the pilicide binding modes observed in the crystal structures of the pilicide in the complex with a free PapD chaperone or the PapD-PapH complex [23,24]. Although, pilicide interactions with conserved I93, located at the end of the β-strand F1, with L32 and with the V56 patch are preserved in these two structures, the electrostatic interactions between R96, located within the loop F1-G1, and R58 residues and carboxyl and carbonyl groups of pilicide are broken as a consequence of the PapH binding to the PapD [24]. The important differences in the structure of the F1-G1 hairpin and the mechanism of DSC reaction observed between the FGS and FGL assembly systems might potentially affect pilicide binding. This gives rise to the question as to whether pilicides that were originally designed on the basis of the structure of the FGS-type PapD and FimC chaperones and were evaluated as inhibitors of the biogenesis of the P and type 1 pili are also active in respect of the FGL assembly pathway.

In this study, we addressed a question denoting the activity of pilicides as inhibitors of the assembly of the Dr fimbriae encoded by the *dra* operon of uropathogenic *E. coli* - the model of the FGL-type adhesive structures [25,26]. These organelles are homopolymers of a single DraE subunit, the structure of which has three receptor binding sites interacting with the following host-cell molecules: Dr^a^ blood-group antigen presented on the CD55/decay-accelerating factor (DAF), the carcinoembryonic antigen (CEA)-related cellular adhesion molecules and the 7S domain of basement membrane protein type IV collagen [27-29]. The assembly of Dr fimbriae is dependent on the action of the DraB chaperone and the DraC usher [17]. The data presented in this article are also important from the epidemiology point of view, as uropathogenic *E. coli* Dr^+^ strains are responsible for 20–25% of cases of cystitis and 30% of pyelonephritis in pregnant woman [30].

## Methods

### General synthesis of pilicides

The reagents were purchased from Sigma-Aldrich. The analytical TLC was performed on aluminum sheets of silica gel UV-254 (Merck). The flash chromatography was carried out using Zeochem silica gel with particle size of 40–63 microns. The NMR spectra ^1^H and ^13^C were recorded at Varian Gemini 200 and Varian Unity Plus 500 in CDCl_3_ or DMSO. The melting points are uncorrected. Pilicides **1** and **2** were prepared in accordance with previously published procedures [22,31]. Given the poor water solubility of the acidic forms of these pilicides, their lithium salts were used in all the experiments. The resulting solutions of compounds were frozen and lyophilized. In order to conduct the experiments, the pilicides were initially dissolved in pure DMSO and the final concentration of DMSO in the growth media was 5%.

### Statistical analysis

In the case of *E. coli* Dr^+^ strain adherence to CHO cells assay and collagen binding assay the statistical significance of results was tested using one-way ANOVA (p-value threshold = 0.05). Influence of pilicides **1** and **2** concentration on the bacterial adherence to CHO cells was assessed relatively to positive control means experiments with adherence of BL21DE3/pBJN406 strain cultivated without pilicide to CHO-DAF^+^ cells. Influence of pilicide **1** concentration on bacterial binding to the polystyrene microtitre plates coated with type IV collagen was assessed relatively to positive control means experiments with BL21DE3/pBJN406 strain cultivated without pilicide.

### Bacterial strains and plasmids

The following *E. coli* strains were used: BL21DE3/pBJN406 – the strain encoding within the pBJN406 plasmid the wild type *dra* operon from the clinical UPEC IH11128 strain, the plasmid is a derivative of the pACYC184 vector; BL21DE3/pACYC184 – a strain used as Dr-type, non-fimbriated, negative control [26,32]. In order to select for the presence of these plasmids, bacteria were grown on media supplemented with chloramphenicol at a concentration of 34 μg/ml.

### Assay of *E. coli* Dr^+^ strain adherence to CHO cells

CHO cells (Chinese hamster ovary K-1) and CHO-DAF^+^ cells stably transfected with cDNA for human DAF [33] were cultured in Ham’s F12 medium supplemented with 10% (vol/vol) fetal bovine serum (Sigma) and a penicillin-streptomycin solution (Sigma) in a 5% CO_2_ atmosphere at 37°C. The cell lines were passaged using 0.25% (vol/vol) trypsin containing EDTA (Sigma). For the adherence assay, the CHO-DAF^+^ and the CHO-DAF^-^ cells were split into 6-well plates with glass coverslips, and grown in the appropriate medium for 18 h. Before the assay, the CHO cells were washed twice with phosphate buffered saline (PBS) and incubated with fresh medium, without antibiotics and without FBS for 1 h. The *E. coli* BL21DE3/pBJN406 strain was cultivated with shaking in Luria-Bertani (LB) medium, supplemented with chloramphenicol, for 24 h at 37°C. 100 μl of the bacterial culture was then split on TSA (trypticase soy agar) plates containing 5% DMSO, chloramphenicol and either supplemented or not with 0.5, 1.5, 2.5 and 3.5 mM pilicide **1** and **2** for another 24 h at 37°C. As the negative control the *E. coli* BL21DE3/pACYC184 strain cultivated on TSA plates not supplemented with pilicides was used. The overnight bacterial strains were harvested from plates washed twice with PBS and resuspended in this buffer to a final OD_600_ of 1.5. 50 μl of each of the *E. coli* bacterial strain suspensions were added to the 4 wells containing the CHO-DAF^+^ or CHO-DAF^-^ cells grown on glass coverslips and incubated for 1.5 h at 37°C. The wells were then washed three times with PBS, fixed with 70% methanol and stained with 10% Giemsa in order to visualize the bound bacteria. Finally, the glass coverslips were examined for bound bacteria under an Olympus inverted microscope (CKX41) with phase-contrast objective. From each coverslip 40 CHO cells were examined and associated bacteria were counted. For each combination of the bacterial strain and CHO cell culture three independent experiments were carried out. To avoid experimenter random errors each experiment was performed using fresh bacterial transformants, fresh CHO cells cultures and fresh preparation of growth media. In all experiment for each combination of the bacterial strain and CHO cell culture four replicates were performed. As a result for each analyzed combination set of twelve data were obtained and analyzed statistically. The obtained values of adherences are expressed as the percentage of mean value of adherence present relative to the CHO-DAF^+^ positive control assay, with a standard deviation because in this form they are more meaningful and easier to compare with the published data.

### Haemagglutination assay

The bacteria were cultivated on TSA plates either supplemented or not with 3.5 mM pilicide, in exactly the same way as for the CHO cells’ adherence assay. The bacteria were scraped from the plates, washed and suspended in PBS buffer to a final OD_600_ of 1.0. These bacterial preparations were used in haemagglutination assays in order to evaluate their level of fimbriation. The human erythrocytes were prepared from blood group O, the whole blood having been donated by a healthy volunteer. The erythrocytes were washed three times with PBS and then suspended in a PBS containing 2% D-mannose to a final OD_640_ of 1.4. The serial dilutions of the bacteria were prepared on 12-well microtitre plates. The mannose resistant haemagglutination (MRHA) assay was performed by adding an equal volume of the erythrocyte suspension to the wells containing bacterial serial dilutions. The haemagglutination experiments were conducted on ice. The last well containing agglutination was visually determined. The HA-titer denotes the inverse of the latest bacterial dilution which still provides agglutination. To confirm that the agglutination observed is an effect of the interaction between the Dr fimbriae and DAF receptor, the reversibility of this reaction as a consequence of chloramphenicol addition to a 2 μM final concentration was monitored. The HA-titers were an average determined from duplicate runs in three independent experiments.

### Collagen binding assay

The wells of the polystyrene microtitre plate were coated with type IV collagen from human placenta (Sigma) at a concentration of 20 mg/ml and incubated at 4°C overnight. They were then washed three times with PBS and blocked with 1% BSA in PBS for 2 h at 37°C. After the removal of the blocking buffer, 100 μl of bacterial cells suspended in a PBS adjusted to a final OD_600_ of 1.0 were added to the collagen-coated coverslips and incubated for another 2 h at 37°C. Additionally, the bacterial preparations were diluted 1:1, 1:2, 1:4, 1:6 and 1:8 in PBS. The bacteria used in the assay were cultivated overnight with shaking in the LB medium (5% DMSO, chloramphenicol), either supplemented or not with 0.5, 1.5, 2.5 and 3.5 mM pilicide **1** for 24 h at 37°C. The Dr fimbriae of the bacteria bound to the collagen were detected with rabbit polyclonal anti-Dr (Immunolab, Poland) and goat anti-rabbit IgG-HRP (Sigma) antibodies at dilutions of 1:500 and 1:5000, with incubation for 40 min at 37°C, respectively. All the antibodies were diluted in a PBS containing 0.2% BSA. The bound antibodies were quantified using Sigma Fast o-phenylenediamine substrate (Sigma) as per manufacturer’s instructions, and measured in an ELISA plate reader (Victor3V, PerkinElmer) at a 490 nm wavelength. The experiment was performed at least three times in duplicate using fresh bacterial transformations and the mean value with standard deviation was determined.

### Densitometry analysis of SDS-PAGE resolved fimbrial fractions

Dr fimbrial fractions were isolated from *E. coli* BL21DE3/pBJN406 grown for 24h on TSA plates (5% DMSO, chloramphenicol) in the presence of 0, 0.5, 1.5, 2.5 and 3.5 mM pilicides **1** and **2**. As a control experiment, a fimbrial fraction was isolated from a non-fimbriated BL21DE3/pACYC184 strain cultivated without pilicide. The bacterial cells were centrifuged (14,000xg), resuspended in a PBS to OD_600_ of 1.0 and vigorously vortexed for 15 min at ambient temperature. The cellular suspensions were then centrifuged (14,000xg) and the supernatants containing the bacterial fimbrial fractions were collected and stored at 4°C. The same volumes (20 μl) of analyzed samples were mixed with Laemmli sample buffer (5 μl), denatured at 100°C for 60 min and ran in 15% (w/v) bis-acrylamide gels containing SDS. To ensure that all the Dr fimbriae were denatured to a monomeric DraE protein, a parallel Western blotting with rabbit anti-Dr serum was conducted. The proteins separated by gel electrophoresis were visualized using Coomasie blue staining. The relative concentration of DraE protein in the fimbrial fractions was determined by means of a densitometry analysis conducted with an SDS-PAGE low-molecular-weight calibration kit (GE Healthcare, Little Chalfont, UK) as a standard, using a VersaDoc system with Quantity One software (both from Bio-Rad, Hercules, CA). The reference *E. coli* BL21DE3/pBJN406 grown without pilicide arbitrary was set to 100%. The experiment was performed three times using fresh bacterial transformations. The summated optical density for the average of the analyzed bands was densitometrically determined from the three measurements for each experiment. For the technical reasons only three experiments were performed and the standard deviation of the mean value was not determined.

## Results

### Rationale for the choice of pilicides

**Figure 1:**
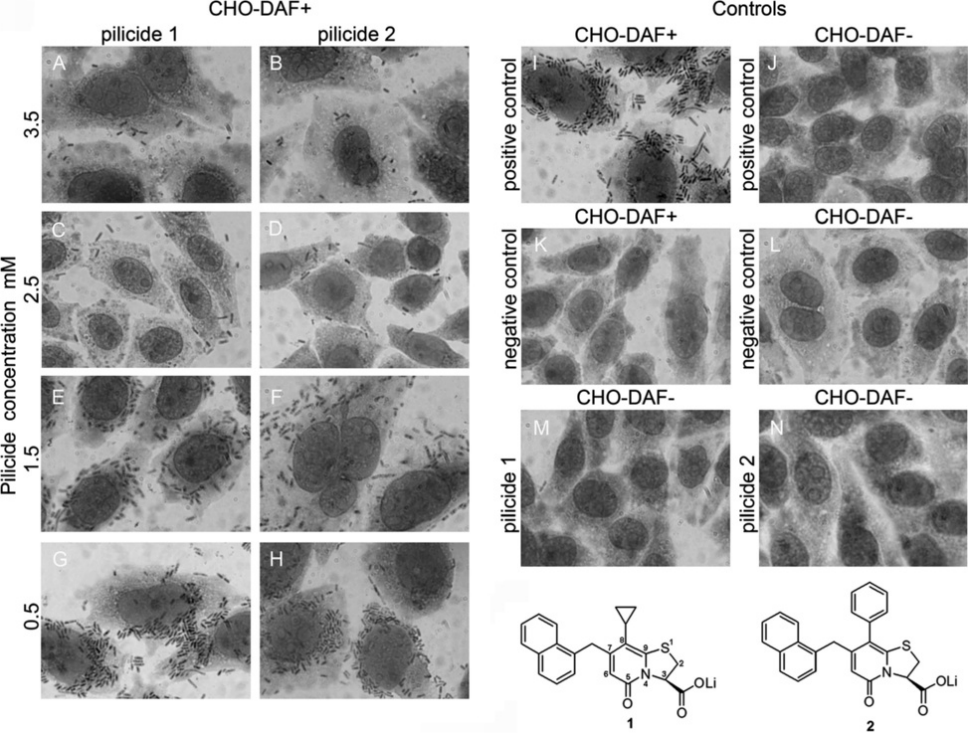
**Blocking the adherence of *E. coli *Dr**^**+ **^**strain to CHO-DAF**^**+ **^**cells by pilicides.** The propensity of bacteria binding to CHO-DAF^+^ and CHO-DAF^-^ cells was evaluated by staining with Giemsa (magnification x 10 000, Olympus CKX41 microscope). The following bacterial preparations were used in the adherence assays: negative control – *E. coli* BL21DE3/pACYC184, grown on TSA plates with 5 % DMSO, non-fimbriated strain; positive control - *E. coli* BL21DE3/pBJN406, grown on TSA plates without pilicide, fully-fimbriated strain; pilicide **1** and pilicide **2** - *E. coli* BL21DE3/pBJN406 grown on TSA plates in the presence of 0.5, 1.5, 2.5 and 3.5 mM of agents **1** and **2**, respectively. In the experiments presented by M and N panels the *E. coli* BL21DE3/pBJN406 strain was grown in the presence of 3.5 mM of pilicides. The figure presents representative results obtained from three independent experiments. Each experiment was composed from the four-fold repetition for each used bacterial preparation. The bacterial adherence to 40 CHO cells was determined for each repetition. Presented in the figure pilicides **1** and **2** are the literature agents which, at a 3.5 mM concentration, inhibit the assembly of FGS type 1 and P pili.

To evaluate the potential of pilicide activity as blockers of Dr fimbriae biogenesis, we used the published, di-substituted 2-pyridones **1** and **2** (Figure 1) [22,31]. Pilicides **1** and **2** are derivatives of 2-pyridone with CH_2_-1-naphthyl substituent at C-7 and cyclopropyl or phenyl at C-8 position, respectively. The following aspects gave rise to the choice of compounds **1** and **2** for our studies: 1) These compounds belonging to the first generation of pilicides are the most potent inhibitors of P and type 1 pili biogenesis and were thus considered as lead compounds for further structural modifications [34]; 2) There are many data describing activity of these compounds as blockers of P and type 1 pili assembly including biological assays on whole bacterial cells, *in vitro* evaluation of pilicide affinity to the chaperone molecules and crystallographic data describing pilicide binding to the chaperone [21,23,24,34-36]; and 3) The pilicides described so far were originally constructed and subsequently modified on the basis of structural data describing the PapD and FimC chaperones [22]. The use of lead compounds **1** and **2** with undecorated C-2 and C-6 positions in experiments should give more general results on pilicide activity against FGL-type adhesive organelles. In our studies evaluating the anti-microbial activity of pilicides **1** and **2** as potential inhibitors of Dr fimbriae biogenesis, we conducted whole bacteria cell experiments because, in contrast to *in vitro* protein – ligand assays, they generate more relevant biological data. We used *E. coli* BL21DE3 strain transformed with pBJN406 plasmid carrying the wild type *dra* gene cluster in the experiments. This strain is routinely used as the laboratory model of the clinical UPEC strain IH11128 from which the *dra* operon was isolated [26,32]. For most *in vivo* experiments, the activity of pilicides **1** and **2** as inhibitors of type 1 and P pili formation was determined for the 3.5 mM pilicide concentration. In order to perform a straight comparison with the published data, we primarily analyzed the influence of pilicides on the Dr fimbriae biogenesis using the 3.5 mM concentration and exposed these data in the text. At this concentration, the pilicides exerted a statistically unimportant effect on the bacterial growth in comparison to the strain cultivated without pilicide. The pilicides **1** and **2** were produced in accordance with literature procedures [22,31].

### Pilicides block Dr fimbriae-dependent bacterial adherence

At the first stage, we determined the adherence of bacteria cultivated on TSA plates in the presence of 0.5, 1.5, 2.5 and 3.5 mM of pilicides **1** and **2** to the CHO cells transfected with plasmid encoding DAF receptor protein recognized by Dr fimbriae. The process of bacteria attachment was visualized by means of Giemsa staining. In the case of strain BL21DE3/pBJN406 cultivated without pilicide (positive control), we observed a high level of bacteria attachment to the CHO-DAF^+^ cells related to the undisturbed production of Dr fimbriae (Figure 1I). The adherence of positive control is set as 100% ±12 and the observed adherences of all other used bacterial preparations are expressed as the percentage of mean value of adherence present relative to control. The addition of 3.5 mM pilicide to the bacterial growth media resulted in a very high reduction in the bacterial adhesion properties: for pilicide **2**, only a few bacterial cells were visible as attached, corresponding to the relative bacterial adherence of 13% ±3 (Figure 1B) and for pilicide **1** resulted in a slightly lower inhibition of bacterial attachment, corresponding to the relative adherence of 25% ±7 (Figure 1A). *E. coli* BL21DE3/pBJN406 bacterial strains cultivated in the presence of 0.5, 1.5 and 2.5 mM of pilicides **1** and **2** showed dose dependent relative adherence of: 90% ±3, 60% ±5 and 32% ±6 for pilicide **1** and 92% ±8, 42% ±7 and 21% ±9 for pilicide **2**, respectively (Figure 1 G,E,C and H,F,D). In order to confirm that the bacterial adherence is dependent on the specific interactions between the DraE fimbrial subunits and DAF, we used as the control non-transfected CHO cells, which do not express DAF molecules naturally. The relative adherence of Dr-fimbriated BL21DE3/pBJN406 positive control (Figure 1J), non-fimbriated BL21DE3/pACYC184 negative control (Figure 1L) and BL21DE3/pBJN406 strain grown in the presence of 3.5 mM of pilicide **1** or **2** (Figure 1M and N) to the CHO-DAF^-^ cells for all experiments was of 3-6% ± 1–2. The similar value of relative adherence of 5% ±6 was determined for binding of non-fimbriated BL21DE3/pACYC184 negative control strain to CHO-DAF^+^ cells. Statistical analysis confirmed the significance of decreased adherence (p < 0.05) after the exposure of bacteria on different concentration of pilicides. Only the result for the lowest concentration of pilicide **2** was statistically not significant relatively to the positive control (p = 0.068). The increasing concentration of pilicides also had the influence on adhesion level (p < 0.05).

**Figure 2:**
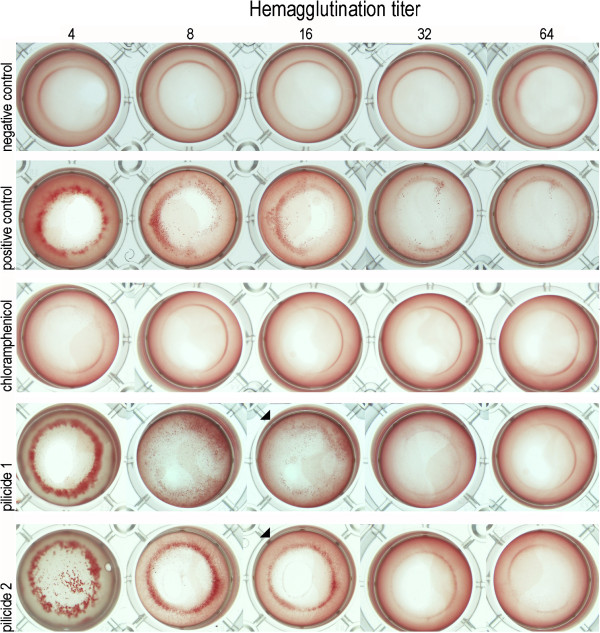
**Blocking of Dr fimbriae-dependent agglutination of human erythrocytes by pilicides.** The following bacterial preparations, normalized to OD_600_, were used in the hemagglutination assays: negative control – *E. coli* BL21DE3/pACYC184, grown on TSA plates with 5% DMSO, non-fimbriated strain; positive control - *E. coli* BL21DE3/pBJN406, grown on TSA plates without pilicide, fully-fimbriated strain; chloramphenicol –*E. coli* BL21DE3/pBJN406, grown on TSA plates without pilicide, the agglutination experiment was performed in the presence of 2 μM of chloramphenicol; pilicide **1** and pilicide **2** - the *E. coli* BL21DE3/pBJN406 grown on the TSA plates in the presence of 3.5 mM of agents **1** and **2**, respectively. HA titer represents two-fold serial dilutions of normalized bacterial suspensions. The initial 1, 2 and final 128 dilutions are not presented. In the case of HA assays with bacteria cultivated in media in the presence of pilicide the black triangles mark the highest dilution which still provides visible agglutination.

For further evaluation of the activity of compounds **1** and **2** as inhibitors of Dr fimbriae biogenesis, we used a haemagglutination test (HA) conducted in a manner similar to the case of published data describing the activity of pilicides **1** and **2** as P and type 1 pili biogenesis inhibitors [34]. The assay is based on an analysis of human erythrocyte agglutination mediated by the bacterial cells. The reaction is dependent on the specific interaction between Dr fimbriae and DAF receptor located on the erythrocyte surfaces. The interaction between DAF receptor and Dr fimbriae is inhibited by the addition of chloramphenicol at a concentration of 2 μM [37]. The specificity of the haemagglutination observed was confirmed by an analysis of its reversibility as a consequence of the addition of chloramphenicol. The observed haemagglutinating ability of the bacteria reflects the amount of Dr fimbriae produced in the presence of the pilicide. The HA-titer, the highest bacterial dilution, in duplicates, which still provides erythrocyte agglutination is determined in the experiment (Figure 2). A low HA-titer indicates that a higher concentration of bacteria, with low amount of fimbriae, is required for agglutination to occur. In our assay, the bacteria of *E. coli* BL21DE3/pBJN406 were grown analogically to the CHO cells adherence experiments, on agar plates containing 3.5 mM pilicide. The fully-fimbriated bacteria grown in the absence of pilicide (positive control) resulted in an HA-titer of 128. The non-fimbriated bacteria *E. coli* BL21DE3/pACYC184 (negative control) gave an HA-titer of 1. The bacteria cultivated in the presence of pilicides **1** and **2** in media had a reduced HA-titer of 16/32 (Figure 2). The HA-titers were determined as an average from duplicate runs in three independent experiments These results clearly show that bacteria grown in the presence of these pilicides possess a reduced amount of Dr fimbriae as an effect of blocking the chaperone-usher pathway.

### Pilicide-treated bacteria possess a reduced quantity of Dr fimbriae

**Figure 3:**
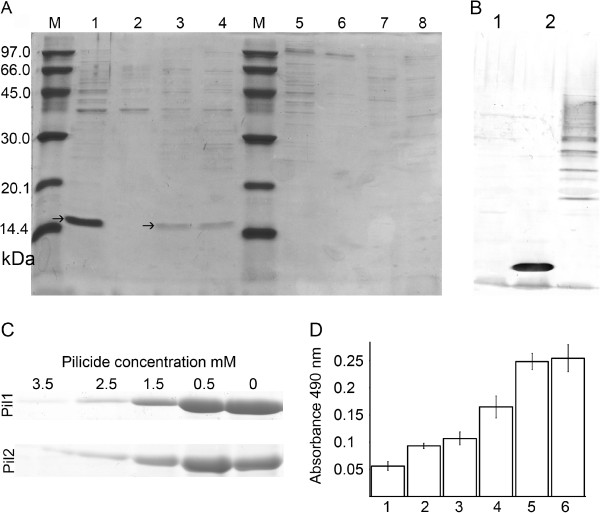
**Relative determination of Dr fimbriae amount on bacteria treated with pilicides.** (**A**) SDS-PAGE analysis of the fimbrial fractions isolated from the following bacterial cultures: lanes 1,5 - BL21DE3/pBJN406, grown on TSA plates without the pilicide, fully-fimbriated strain; 2,6 - BL21DE3/pACYC184, non-fimbriated strain; 3,7 and 4,8 - BL21DE3/pBJN406, grown in the presence of 3.5 mM of agents **1** and **2**, respectively. Before electrophoresis, the samples from 1 to 4 and from 5 to 8 were incubated for 60 min at 100°C and 25°C, respectively. M – the SDS-PAGE LMW Calibration Kit weight standard. Arrow denoted monomeric DraE protein. (**B**) Western blotting analysis of the fimbrial fractions, performed to confirm the complete depolymerization of Dr fimbriae during sample denaturation. 1,2,3 – the same samples as in lanes 2,1 and 5 in panel B, respectively. (**C**) SDS-PAGE analysis of fimbrial fractions isolated from *E. coli* BL21DE3/pBJN406 grown on TSA plates supplemented with different concentrations of pilicide **1** (Pil1) and pilicide **2** (Pil2). Densitometrically evaluated DraE amounts are expressed as the percentage of mean value present relative to bacterial strain cultivated without pilicide, arbitrary set to 100%: 0.5 mM – 92 and 97%; 1.5 mM – 45 and 55%; 2.5 mM – 32 and 25%; 3.5 mM – 25 and 20% for strains grown in the presence of pilicide **1** and **2**, respectively. (**D**) Evaluation of bacteria fimbriation using an ELISA assay with microtitre plates coated with type IV human collagen. The Dr fimbriae exposed on the bacteria adhered to collagen were visualized using anti-Dr antibodies. The following bacterial preparations were used in the assay: 1 - BL21DE3/pACYC184, non-fimbriated strain; 2-5 - BL21DE3/pBJN406, grown in LB medium supplemented with 3.5, 2.5, 1.5 and 0.5 mM of agent **1**, respectively; 6 - BL21DE3/pBJN406, grown in LB medium without the pilicide, fully-fimbriated strain. The bars represent the s.d. of the mean of three independent experiments in duplicate.

In order to monitor the effect of pilicides on the volume of Dr fimbriae production quantitatively, we used two indirect assays; an ELISA, with anti-Dr antibodies, and a densitometry analysis of fimbrial fractions resolved by SDS-PAGE. Apart from interacting with DAF, the Dr fimbriae also recognize type IV collagen as a receptor. In the ELISA the wells of the polystyrene microtitre plate were coated with type IV human collagen. After the blocking step, different dilutions of bacteria were added and the amount of Dr fimbriae was detected using rabbit anti-Dr and anti-rabbit IgG-HRP antibodies. The bacteria *E. coli* BL21DE3/pBJN406 and BL21DE3/pACYC184 were grown in Luria-Bretani media because the assays performed on bacteria scraped from agar result in a high background during an ELISA test. Pilicide activity was only evaluated for compound **1** at the concentration 0.5, 1.5, 2.5 and 3.5 mM, as pilicide **2** precipitates in LB medium containing 5% DMSO during cultivation. In experiments, the amount of Dr fimbriae for strain *E. coli* BL21DE3/pBBJN406 grown in the presence of 0.5, 1.5, 2.5 and 3.5 mM pilicide **1** was reduced by 3%, 45% 74% and 81%, respectively in relation to the same bacteria grown without pilicide (Figure 3D). Decreasing of Dr fimbriae amount caused only by 0.5 mM pilicide dilution was not statistically significant (p = 0.625), higher concentrations provided p-value much below 0.05. Also increasing concentration of pilicides was statistically significant for Dr fimbriae amount reduction (p < 0.05).

To further evaluate the effect of pilicides on the inhibition of Dr fimbriae production, we quantified the amount of monomeric DraE protein resulting from the denaturation/depolimerization of isolated Dr fimbriae samples using a densitometric analysis of the SDS-PAGE gels stained with Coomassie Blue (Figure 3A-C). The strain *E. coli* BL21DE3/pBJN406 was grown on agar plates supplemented with pilicides **1** and **2** at a concentration of 0.5, 1.5, 2.5 and 3.5 mM. Non-fimbriated *E. coli* BL21DE3/pACYC184 and fully-fimbriated BL21DE3/pBJN406 strains grown without pilicide were used as the negative and positive controls, respectively. The fimbriae from the bacteria scraped and normalized to OD_600_ were isolated by means of vortexing. Dr fimbriae are very stable structures which require extending heating in Laemmli buffer in order to depolimerize to a monomeric DraE protein. The band of monomeric DraE protein was visible in resolved samples heated for 60 min at 100°C before electrophoresis. In contrast, there was no band corresponding to monomeric DraE in the samples which had not been denatured thermally before electrophoresis (Figure 3A). This confirms that the isolated fractions only contained Dr fimbriae and were not contaminated by the monomeric, periplasmic form of DraE protein. In order to prove that the heating time for the samples is sufficient for the total denaturation of Dr fimbrial structures to monomeric DraE, we performed a Western blotting analysis with anti-Dr antibodies (Figure 3B). In these experiments, the estimated pilicide effects of compounds **1** and **2** were comparable (Figure 3C). For bacteria cultivated in the presence of 3.5 mM of pilicides **1** and **2**, the amount of DraE fimbrial protein was reduced by 75 and 80% in comparison to the fully-fimbriated strain grown without pilicide, respectively. Performing experiments with 0.5, 1.5 and 2.5 mM concentration of pilicides, we analyzed their dose dependent effects on the volume of fimbrial production. At a concentration of 2.5 mM, pilicides **1** and **2** inhibit fimbrial production at comparable level as in the case of bacteria treated with the 3.5 mM concentration, – adherence reduction by 68 and 75%, respectively. The use of pilicides **1** and **2** at a concentration of 1.5 mM results in a relative DraE reduction of 55 and 45%, respectively. Bacteria cultivated with 0.5 of pilicides **1** and **2** have almost the same amount of the DraE protein derived from Dr fimbriae as in the case of strain grown without the addition of the compounds, – adherence reduction 8 and 3%, respectively.

## Discussion

**Figure 4:**
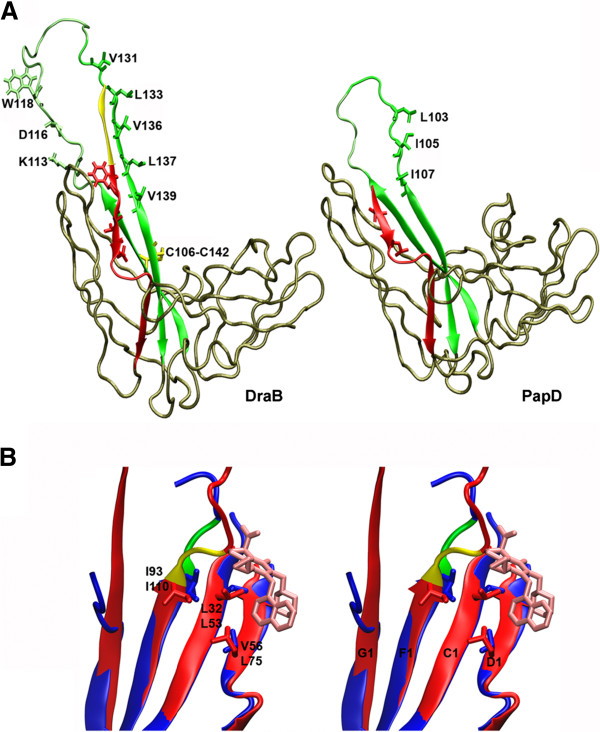
**DraB as a model of the FGL subfamily of chaperones.** (**A**) Characteristic elements of the DraB structure (PDB ID: 4DJM) specific to the FGL chaperones in relation to the PapD (PDB ID: 2WMP), - the representative of the FGS subfamily. The part of β-strand A1 with hydrophobic residues participating in subunit interaction represented in the bonds mode is denoted in red. The fragment of the long N-terminal region of the β-strand A1 characteristic for FGL chaperones observed in the DraB is denoted in yellow. The F1 strand-loop-G1 strand hairpin motif is denoted in green with the alternating hydrophobic residues of the β-strand G1 participating in the DSC reaction denoted in the bonds mode. In the F1-G1 loop of the DraB protein, the conserved residue motif KDW characteristic for FGL chaperones are marked with the bond mode. The disulfide bond binding β-strands F1 and G1 in the DraB structure conserved in the entire FGL subfamily is marked in yellow bond mode. The F1-G1 loop region was modeled using MODELLER v9.2 software. (**B**) Structural alignment of the usher binding site of DraB (red) and PapD-pilicide (PDB ID: 2J7L) (blue) with denoted hydrophobic patch that includes I93, L32, V56 (PapD) and I110, L56, L32 (DraB) residues forming pilicide (pink) binding motif. At the beginning of the F1-G1 loop the region of two proline residues forming “proline lock” conserved in the family of chaperones is denoted (P111 and P112 in the DraB – yellow; P94 and P95 in the PapD - green).

The anti-bacterial activity of pilicides has only been confirmed in the case of uropathogenic *E. coli* producing type 1 and P pili which represent the FGS type organelles. In this paper for the first time we investigated the activity of pilicides as inhibitors of the FGL-type adhesion structure biogenesis using as a model Dr fimbriae. The sequence and structural analyses of the DraB chaperone (PDB ID: 4DJM) reveal that it possesses all the marks characteristic for the FGL Caf1M-like chaperones (Figure 4A): 1) the β-strands F1 and G1 are connected by the long loop, which is composed of 15 residues; 2) the G1 donor strand contains five bulky hydrophobic residues; 3) the N-terminal subunit binding motif including the β-strand A1 with three bulky hydrophobic residues is very long and contains 26 residues, whereas the PapD has only 7 residues, 2 of them bulky hydrophobic; 4) the conserved disulfide bond stabilizes a massive F1-loop-G1 hairpin; 5) the three conserved residues, namely, K105, D107 and W110, are located in the loop connecting the β-strands F1 and G1 [12,13]. The X-ray structures published showing the structure of a pilicide interacting with the free PapD chaperone revealed that the ligand affects the hydrophobic patch located in the F1-C1-D1 β-sheet of the N-terminal domain formed by the residues I93, L32 and V56 [23,24]. An homologous motif, which could, presumably be a pilicide binding site, is also present in the structure of the DraB chaperone and encompasses residues L53, L75 and I110. The geometry of this region is very similar to that observed in the PapD protein (Figure 4B). The structural analysis of DraB allows us to treat it as a model representative of a sub-family of the FGL-like chaperones.

Activity of pilicides **1** and **2** as inhibitors of Dr fimbriae biogenesis was tested on the *E. coli* BL21DE3/pBJN406 – the laboratory model of the clinical UPEC IH11128 strain. Biological evaluations based on the whole-cell assays were predominantly performed using a 3.5 mM concentration of pilicides, as is used in the case of most experiments with an inhibition of type 1 and P pili formation. The *E. coli* BL21DE3/pBJN406 bacteria cultivated in the presence of 3.5 mM pilicides **1** and **2** showed the amount of DraE subunits/Dr fimbriae reduced by 75–80% as determined by SDS-PAGE densitometry analysis of isolated fimbrial fractions. A Western immunoblot analysis of this strain with anti-Dr antibodies denoted a reduction, by 81%, of the amount of Dr fimbriae in relation to fully-fimbriated, pilicide untreated bacteria. The amounts of major pili P PapA (recombinant strain HB101/pPAP5) and type 1 pili FimA (clinical strain UTI89) subunits isolated from bacteria cultivated in the presence of 3.5 mM of pilicide **1** analyzed by immunoblot were reduced by 68% and 53%, respectively [23,36]; in the case of FimA, the C-6 morpholinomethyl substituent in pilicide **1** with no effects on its biological activity was compared. The atomic force microscopy analysis of the HB101/pPAP5 strain showed that the bacteria treated with 3.5 mM of pilicide **1** were devoid of P pili [36]. The inhibition of Dr fimbriae production by 3.5 mM pilicides **1** and **2** is reflected in the 25% ± 7 and 13 ±3% DAF dependent bacteria relative adherence to CHO cells, respectively. This correlates well with the 90% reduction in adherence to the bladder cells of *E. coli* NU14 producing type 1 pili cultivated in the presence of a C-6 morpholinomethyl derivative of pilicide **1**[23]. In the haemaglutynation assay, which also reflects the adherence properties of *E. coli* BL21DE3/pBJN406 Dr^+^ strain treated with 3.5 mM pilicides **1** and **2**, we observed an HA-titer of 16/32; the strain untreated with pilicide constituting the control has an HA-titer of 128. Published HA-titer data for the HB101/pPA5A strain, treated and untreated with pilicide **1**, are 1/4 and 128, respectively [34,36]. The difference observed in the HA-titer of the pilicide-treated Dr- and pili P-producing strains is difficult to analyze because of experimental reasons. We only presumed that the higher haemagglutination properties of Dr fimbriae-producing bacteria might be connected with the polyadhesin nature of these structures, in contrast to the monoadhesin of P pili. As the DraE subunits are multi-receptor adhesins, the inhibition of Dr fimbriae assembly by pilicides was also confirmed by the evaluation of bacterial adherence to the type IV human collagen receptor. The SDS-PAGE analysis of isolated fimbrial fractions, collagen binding assay and Dr fimbriae dependent bacterial adherence to CHO-DAF^+^ cells assay performed using bacteria cultivated in the 0, 0.5, 1.5, 2.5 and 3.5 mM of compounds **1** and **2** confirmed that the effect of Dr fimbriae assembly inhibition observed was dependent on the pilicide concentration used. This is a crucial feature of the antibacterial agents. The data based on the whole cell assays presented in this article confirm that pilicides effectively inhibit the receptor-dependent adherence of *E. coli* Dr^+^ strain to the host cells. Thus pilicides impair the crucial step of bacterial pathogenesis, namely, – the formation of initial, close contact between bacteria and host cell. The evaluations of the pilicides’ effects on *E. coli* Dr^+^ strain are comparable to those previously published for type 1- and P pili-producing bacteria. This suggests that the structural and functional differences observed between FGS and FGL chaperone-usher systems are not crucial to pilicide activity. This thesis is supported by the structure of the Caf1-Caf1M subunit-chaperone pre-assembly complex bound to the N-terminal domain of Caf1A usher – the example of the FGL system [11]. Although Caf1A and FimD belong to the FGL and FGS subfamilies of usher respectively, their N-terminal domains represents a high degree of structural similarity. The structures of usher binding sites that encompass pilicide binding residues are also highly conserved in the FGL and FGS type chaperones (Figure 4B). Comparison of the free Caf1M and Caf1-Caf1M complex structures permits to identify in the usher binding site of Caf1M chaperone specific “proline lock” that by interaction with Caf1 subunit allostericaly controls the chaperone-usher pathway [11]. Such ”proline lock” was also identified in the available sequences and structures of usher binding sites of the other FGS and FGL type chaperones including DraB (Figure 4B) [11]. This clearly shows that interaction between N-terminal domain of usher and usher binding motif of chaperones is highly conserved structurally and mechanically.

## Conclusions

We conclude that pilicides **1** and **2** in mM concentration effectively inhibit the adherence of the laboratory model of uropathogenic *E. coli* Dr^+^ strain, - the main causative agent of cystitis and pyelonephritis in pregnant women, to the host cell DAF and collagen receptors by blocking the assembly of Dr fimbriae. On the basis of the high conservation of the Dr family of adhesins, which include clinically important human UPEC strains, we deduce that the pilicides should also effectively inhibit their assembly and, more speculatively, other FGL adhesive structures. The earlier thesis proposed by pilicide originators: “Pilicides, by blocking chaperone and usher function, have the potential to inhibit pili formation in a broad spectrum of pathogenic bacteria to prevent critical host-pathogen interactions necessary for many diseases [23]” has been considerably reinforced experimentally by extending the examination of pilicide activity from FGS-type structures to the assembly of FGL-type Dr fimbriae.

## Competing interests

The authors declare that they have no financial and non-financial competing interests.

## Authors’ contributions

RP designed and coordinated the project, performed the experimental data analysis and wrote the manuscript. BZP performed the assays of *E. coli* Dr^+^ strain adherence to CHO cells and the ELISA-based, collagen binding assay. ACC implemented the physicochemical methods and statistical analysis of the data. SM and KD performed the chemical synthesis of the pilicides. JP performed the hemagglutination assays and the SDS-PAGE procedures. KS performed the statistical analysis of data. MW carried out the structural analysis of DraB chaperone. All the authors read and approved the final manuscript.

## References

[B1] Justice SS Hung C Theriot JA Fletcher DA Anderson GG Footer MJ Hultgren SJ Differentiation and developmental pathways of uropathogenic Escherichia coli in urinary tract pathogenesis Proc Natl Acad Sci USA 2004 101 1333 1338 10.1073/pnas.0308125100 14739341 PMC337053

[B2] Wright KJ Seed PC Hultgren SJ Development of intracellular bacterial communities of uropathogenic Escherichia coli depends on type 1 pili Cell Microbiol 2007 9 2230 2241 10.1111/j.1462-5822.2007.00952.x 17490405

[B3] Sauer FG Futterer K Pinkner JS Dodson KW Hultgren SJ Waksman G Structural basis of chaperone function and pilus biogenesis Science 1999 285 1058 1061 10.1126/science.285.5430.1058 10446050

[B4] Choudhury D Thompson A Stojanoff V Langermann S Pinkner J Hultgren SJ Knight SD X-ray structure of the FimC-FimH chaperone-adhesin complex from uropathogenic Escherichia coli Science 1999 285 1061 1066 10.1126/science.285.5430.1061 10446051

[B5] Zavialov AV Berglund J Pudney AF Fooks LJ Ibrahim TM MacIntyre S Knight SD Structure and biogenesis of the capsular F1 antigen from Yersinia pestis: preserved folding energy drives fiber formation Cell 2003 113 587 596 10.1016/S0092-8674(03)00351-9 12787500

[B6] Zavialov AV Tischenko VM Fooks LJ Brandsdal BO Aqvist J Zav'yalov VP Macintyre S Knight SD Resolving the energy paradox of chaperone/usher-mediated fibre assembly Biochem J 2005 389 685 694 10.1042/BJ20050426 15799718 PMC1180718

[B7] Barnhart MM Pinkner JS Soto GE Sauer FG Langermann S Waksman G Frieden C Hultgren SJ PapD-like chaperones provide the missing information for folding of pilin proteins Proc Natl Acad Sci USA 2000 97 7709 7714 10.1073/pnas.130183897 10859353 PMC16609

[B8] Sauer FG Pinkner JS Waksman G Hultgren SJ Chaperone priming of pilus subunits facilitates a topological transition that drives fiber formation Cell 2002 111 543 551 10.1016/S0092-8674(02)01050-4 12437927

[B9] Remaut H Rose RJ Hannan TJ Hultgren SJ Radford SE Ashcroft AE Waksman G Donor-strand exchange in chaperone-assisted pilus assembly proceeds through a concerted beta strand displacement mechanism Mol Cell 2006 22 831 842 10.1016/j.molcel.2006.05.033 16793551 PMC7617774

[B10] Remaut H Tang C Henderson NS Pinkner JS Wang T Hultgren SJ Thanassi DG Waksman G Li H Fiber formation across the bacterial outer membrane by the chaperone/usher pathway Cell 2008 133 640 652 10.1016/j.cell.2008.03.033 18485872 PMC3036173

[B11] Di Yu X Dubnovitsky A Pudney AF Macintyre S Knight SDAVZ Allosteric mechanism controls traffic in the chaperone/usher pathway Structure 2012 20 1861 1871 10.1016/j.str.2012.08.016 22981947

[B12] Zavialov A Zav'yalova G Korpela T Zav'yalov V FGL chaperone-assembled fimbrial polyadhesins: anti-immune armament of Gram-negative bacterial pathogens FEMS Microbiol Rev 2007 31 478 514 10.1111/j.1574-6976.2007.00075.x 17576202

[B13] Zav'yalov V Zavialov A Zav'yalova G Korpela T Adhesive organelles of Gram-negative pathogens assembled with the classical chaperone/usher machinery: structure and function from a clinical standpoint FEMS Microb Rev 2010 34 317 378 10.1111/j.1574-6976.2009.00201.x 20070375

[B14] Roy SP Rahman MM Yu XD Tuittila M Knight SD Zavialov A Crystal structure of enterotoxigenic Escherichia coli colonization factor CS6 reveals a novel type of functional assembly Mol Microbiol 2012 86 1100 1115 10.1111/mmi.12044 23046340

[B15] Hung DL Knight SD Woods RM Pinkner JS Hultgren SJ Molecular basis of two subfamilies of immunoglobulin-like chaperones EMBO J 1996 15 3792 3805 8670884 PMC452060

[B16] Zav'yalov VP Zav'yalova GA Denesyuk AI Korpela T Modelling of steric structure of a periplasmic molecular chaperone Caf1M of Yersinia pestis, a prototype member of a subfamily with characteristic structural and functional features FEMS Immunol Med Microbiol 1995 11 19 24 10.1111/j.1574-695X.1995.tb00074.x 7599600

[B17] Piątek R Zalewska B Kolaj OMF Nowicki B Kur J Molecular aspects of biogenesis of Escherichia coli Dr Fimbriae: characterization of DraB-DraE complexes Infect Immun 2005 73 135 145 10.1128/IAI.73.1.135-145.2005 15618148 PMC538934

[B18] Zav'yalov VP Chernovskaya TV Chapman DA Karlyshev AV MacIntyre S Zavialov AV Vasiliev AM Denesyuk AI Zav'yalova GA Dudich IV Influence of the conserved disulphide bond, exposed to the putative binding pocket, on the structure and function of the immunoglobulin-like molecular chaperone Caf1M of Yersinia pestis Biochem J 1997 324 571 578 9182720 10.1042/bj3240571 PMC1218468

[B19] Jonson AB Normark S Rhen M Fimbriae, pili, flagella and bacterial virulence Contrib Microbiol 2005 12 67 89 15496777 10.1159/000081690

[B20] Nuccio SP Bäumler AJ Evolution of the chaperone/usher assembly pathway: fimbrial classification goes Greek Microbiol Mol Biol Rev 2007 71 551 575 10.1128/MMBR.00014-07 18063717 PMC2168650

[B21] Aberg V Almqvist F Pilicides-small molecules targeting bacterial virulence Org Biomol Chem 2007 5 1827 1834 10.1039/b702397a 17551629

[B22] Svensson A Larsson A Emtenäs H Hedenström M Fex T Hultgren SJ Pinkner JS Almqvist F Kihlberg J Design and evaluation of pilicides: potential novel antibacterial agents directed against uropathogenic Escherichia coli Chembiochem 2001 2 915 918 10.1002/1439-7633(20011203)2:12<915::AID-CBIC915>3.0.CO;2-M 11948880

[B23] Pinkner JS Remaut H Buelens F Miller E Aberg V Pemberton N Hedenström M Larsson A Seed P Waksman G Rationally designed small compounds inhibit pilus biogenesis in uropathogenic bacteria Proc Natl Acad Sci USA 2006 103 17897 17902 10.1073/pnas.0606795103 17098869 PMC1693844

[B24] Chorell E Pinkner JS Phan G Edvinsson S Buelens F Remaut H Waksman G Hultgren SJ Almqvist F Design and synthesis of C-2 substituted thiazolo and dihydrothiazolo ring-fused 2-pyridones: pilicides with increased antivirulence activity J Med Chem 2010 12 5690 5695 20586493 10.1021/jm100470t PMC2963145

[B25] Anderson KL Billington J Pettigrew D Cota E Simpson P Roversi P Chen HA Urvil P du Merle L Barlow PN An atomic resolution model for assembly, architecture, and function of the Dr adhesins Mol Cell 2004 15 647 657 10.1016/j.molcel.2004.08.003 15327779

[B26] Nowicki B Barrish JP Korhonen T Hull RA Hull SI Molecular cloning of the Escherichia coli O75X adhesin Infect Immun 1987 55 3168 3173 2890586 10.1128/iai.55.12.3168-3173.1987 PMC260044

[B27] Berger CN Billker O Meyer TF Servin AL Kansau I Differential recognition of members of the carcinoembryonic antigen family by Afa/Dr adhesins of diffusely adhering Escherichia coli (Afa/Dr DAEC) Mol Microbiol 2004 52 963 983 10.1111/j.1365-2958.2004.04033.x 15130118

[B28] Nowicki B Moulds J Hull R Hull S A hemagglutinin of uropathogenic Escherichia coli recognizes the Dr blood group antigen Infect Immun 1988 56 1057 1060 2895740 10.1128/iai.56.5.1057-1060.1988 PMC259761

[B29] Westerlund B Kuusela P Risteli J Risteli L Vartio T Rauvala H Virkola R Korhonen TK The O75X adhesin of uropathogenic Escherichia coli is a type IV collagen-binding protein Mol Microbiol 1989 3 329 337 10.1111/j.1365-2958.1989.tb00178.x 2568575

[B30] Servin AL Pathogenesis of Afa/Dr diffusely adhering Escherichia coli Int J Med Microbiol 2005 295 471 478 10.1016/j.ijmm.2005.07.001 16238021

[B31] Aberg V Hedenström M Pinkner JS Hultgren SJ Almqvist F C-Terminal properties are important for ring-fused 2-pyridones that interfere with the chaperone function in uropathogenic E. coli Org Biomol Chem 2005 3 3886 3892 10.1039/b509376g 16240004

[B32] Väisänen-Rhen V Fimbria-like hemagglutinin of Escherichia coli O75 strains Infect Immun 1984 46 401 407 6150006 10.1128/iai.46.2.401-407.1984 PMC261546

[B33] Lublin DM Coyne KE Phospholipid-anchored and transmembrane version of either decay-avvelerating factor or membrane cofactor protein show equal efficiency in protection from complement-mediated cell damage J Exp Med 1991 174 35 10.1084/jem.174.1.35 1711565 PMC2118896

[B34] Aberg V Sellstedt M Hedenström M Pinkner JS Hultgren SJ Almqvist F Design, synthesis and evaluation of peptidomimetics based on substituted bicyclic 2-pyridones-targeting virulence of uropathogenic E. coli Bioorg Med Chem 2006 14 7563 7581 10.1016/j.bmc.2006.07.017 16904898

[B35] Aberg V Das P Chorell E Hedenström M Pinkner JS Hultgren SJ Almqvist F Carboxylic acid isosteres improve the activity of ring-fused 2-pyridones that inhibit pilus biogenesis in E. coli Bioorg Med Chem Lett 2008 18 3536 3540 10.1016/j.bmcl.2008.05.020 18499455 PMC3665338

[B36] Aberg V Fällman E Axner O Uhlin BE Hultgren SJ Almqvist F Pilicides regulate pili expression in E. coli without affecting the functional properties of the pilus rod Mol Biosyst 2007 3 214 218 10.1039/b613441f 17308668

[B37] Pettigrew D Anderson KL Billington J Cota E Simpson P Urvil P Rabuzin F Roversi P Nowicki B du Merle L High resolution studies of the Afa/Dr adhesin DraE and its interaction with chloramphenicol J Biol Chem 2004 279 46851 46867 10.1074/jbc.M409284200 15331605

